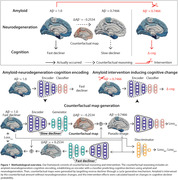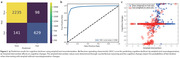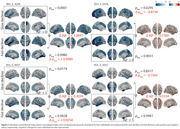# Counterfactual reasoning‐based intervention effect estimation for longitudinal cognitive decline

**DOI:** 10.1002/alz.092108

**Published:** 2025-01-09

**Authors:** Sohyun Kang, Yeong‐Hun Song, Joon‐Kyung Seong

**Affiliations:** ^1^ Korea University, Seoul Korea, Republic of (South); ^2^ NeuroXT, Seoul Korea, Republic of (South)

## Abstract

**Background:**

After the landmark approval of the Aβ‐lowering antibody for treatment of mild cognitive impairment and mild dementia due to Alzheimer’s disease (AD), it has intensified the need to stratify patients based on the likelihood that they will benefit from any amyloid‐lowering treatments currently in the pipeline. We therefore seek to identify individuals most likely to benefit from Aβ‐lowering drugs by estimating intervention effect based on counterfactual reasoning for longitudinal cognitive decline at the individual level.

**Method:**

We utilized 3,542 T1‐weighted magnetic resonance images from the Alzheimer’s Disease Neuroimaging Initiative (ADNI), involving 3,103 Alzheimer’s patients and 439 cognitively normal individuals. Cortical thickness data, processed with FreeSurfer v7.0 and the Desikan‐Killiany atlas, underwent W‐score correction for age, sex, and education to address confounding effect. Amyloid positivity, determined by ADNIMERGE AV45 (>=1.11), was considered. Patients were stratified based on the clinical dementia rating sum of boxes (CDRSB) decline rate into fast or slow decliners. Our framework integrates counterfactual reasoning and intervention effect estimation through a deep explainable AI method rooted in causality. The framework comprises 1) amyloid‐neurodegeneration‐cognition encoding, 2) counterfactual map generation, and 3) intervention effect estimation, illustrated in Figure 1.

**Result:**

The encoder achieved a 5‐fold cross‐validation accuracy of 0.9230 ± 0.0101, with corresponding F1 score and mAUC values of 0.8948 ± 0.0140 and 0.9454 ± 0.0108, respectively (Figure 2). Individual counterfactual neurodegeneration maps targeting slow decliners to fast decliners revealed positive amyloid changes and worsened cognitive outcomes (Figure 3). On the other hands, targeting fast decliners to slow decliners exhibited negative amyloid changes and improved cognitive outcomes. The correlation between cognitive deterioration probabilities (indicating fast decliner status) and amyloid intervention (counterfactual amyloid level) was positive and significant (r=0.2109, p<0.0001) (Figure 2).

**Conclusion:**

Although this work focuses on the fundamental counterfactual reasoning for predicting the rate of longitudinal cognitive decline in the AD spectrum, it also has major potential clinical implications in the era of Aβ‐lowering therapies. By editing the patient‐tailored counterfactual reasoning process, we accurately estimate intervention effect at the baseline timepoint, so that maximize clinical benefit through optimized personalized treatment.